# Severe Phototoxic Vesiculobullous Eruption Following Sequential Exposure to Terbinafine, Ciprofloxacin, and Trimethoprim-Sulfamethoxazole

**DOI:** 10.7759/cureus.109704

**Published:** 2026-05-26

**Authors:** Roberto R Gonzalez Alvarez

**Affiliations:** 1 Hospitalist - Internal Medicine, Cape Coral Hospital, Cape Coral, USA

**Keywords:** adverse drug reaction, bactrim, ciprofloxacin, drug-induced photosensitivity, photo-distributed rash, phototoxic dermatitis, terbinafine, trimethoprim-sulfamethoxazole, vesiculobullous rash

## Abstract

Phototoxic drug eruptions are well-recognized adverse reactions to several classes of medications, including fluoroquinolones, sulfonamides, and antifungals. However, sequential photosensitization following exposure to multiple agents is rarely described. We present the case of a 44-year-old Hispanic male commercial driver who developed a severe, vesiculobullous, photo-distributed rash following sequential exposure to terbinafine, ciprofloxacin, and trimethoprim-sulfamethoxazole (Bactrim). The patient initially presented with ringworm of the lower extremity and was started on topical and oral terbinafine. When the rash worsened approximately one week later, he was sequentially prescribed ciprofloxacin and then Bactrim for suspected cellulitis. Each new medication was associated with progressive worsening of the rash, which was confined to sun-exposed areas (distal arms, distal legs, neck) and characterized by erythema, pruritus, pain, clear fluid-filled vesicles, and superficial skin peeling. There was no mucosal involvement, no Nikolsky sign, and no systemic symptoms. Laboratory evaluation revealed elevated C-reactive protein (2.7 mg/dL) with normal complete blood count, comprehensive metabolic panel, erythrocyte sedimentation rate, and negative autoimmune serologies (antinuclear antibody, anti-SSA, anti-SSB). Infectious workup, including HIV, syphilis, hepatitis panel, and blood cultures, was negative. An infectious disease consultant ruled out onychomycosis and cellulitis. All suspected offending medications were discontinued on admission. The patient was treated with intravenous methylprednisolone (40 mg every eight hours for three days), topical clobetasol, and supportive care. By hospital day three, the erythema had resolved completely, the vesicles had substantially decreased, and the pain had resolved. The patient was discharged home on a prednisone taper. This case highlights the importance of recognizing phototoxic drug eruptions in patients presenting with photo-distributed vesiculobullous rashes, particularly when multiple photosensitizing medications have been prescribed sequentially. A systematic approach to excluding infectious, autoimmune, and other drug-induced conditions is essential for diagnosis when a biopsy is not available.

## Introduction

Phototoxic drug eruptions are non-immunologic, dose-dependent cutaneous reactions triggered by the interaction of a photosensitizing medication and ultraviolet (UV) radiation, typically UVA [1-3]. Clinically, phototoxic reactions resemble an exaggerated sunburn, presenting as erythema, edema, pruritus, burning sensation, and in severe cases, vesiculation and bullae formation confined to sun-exposed areas [1,2,4,5]. Unlike photoallergic reactions, phototoxicity can occur on first exposure and does not require prior sensitization [6,7]. Of note, UVA radiation can penetrate standard vehicle side windows, which is particularly relevant for patients with occupational sun exposure, such as commercial drivers.

The photosensitizing properties of each drug class are discussed in detail later. Fluoroquinolones (particularly ciprofloxacin and levofloxacin) are among the most commonly implicated [8,9]. Trimethoprim-sulfamethoxazole (Bactrim) is also a well-documented photosensitizer [1,4]. Terbinafine, an allylamine antifungal, has been increasingly recognized as a cause of photosensitivity, including phototoxic and photoallergic reactions [10,11].

The diagnosis of phototoxic drug eruption is primarily clinical, based on a consistent temporal relationship between drug exposure and symptom onset, photo-distributed rash morphology, and improvement following drug withdrawal and sun avoidance [6,12]. However, the differential diagnosis is broad and includes systemic lupus erythematosus, dermatomyositis, porphyria cutanea tarda, fixed drug eruption, Stevens-Johnson syndrome (SJS), toxic epidermal necrolysis (TEN), and drug reaction with eosinophilia and systemic symptoms (DRESS) syndrome [13-15].

We present a case of severe phototoxic vesiculobullous eruption following sequential exposure to three photosensitizing medications-terbinafine, ciprofloxacin, and trimethoprim-sulfamethoxazole-in a patient with significant occupational sun exposure. This case is noteworthy for the sequential nature of drug-induced worsening and the systematic exclusion of alternative diagnoses.

## Case presentation

Patient information and history

A 44-year-old Hispanic male commercial driver with no significant past medical history presented to the hospital with a progressively worsening, painful, pruritic rash involving sun-exposed areas of his body. He had no known drug allergies.

The patient's clinical course began when he developed symptoms consistent with ringworm (tinea corporis) on his right lower extremity. The initial diagnosis of ringworm was made clinically by the patient's primary care physician; no confirmatory testing (KOH preparation, fungal culture, or dermatologic evaluation) was performed prior to the initiation of terbinafine. He sought medical attention from his primary care physician (PCP) and was prescribed topical terbinafine 1% cream twice daily and oral terbinafine 250 mg daily, both for a planned 14-day course. This was designated as day 0 of the clinical timeline.

Sequential drug exposure and rash progression

Around day seven of terbinafine therapy, the patient noted that his skin rash was worsening rather than improving. The lesion had become larger, more erythematous, and began to spread. He returned to his PCP, who diagnosed cellulitis and prescribed ciprofloxacin 500 mg twice daily for a planned seven-day course. The terbinafine was continued.

Approximately six days after starting ciprofloxacin, the rash continued to worsen. The patient contacted his PCP, who discontinued ciprofloxacin and switched therapy to trimethoprim-sulfamethoxazole (Bactrim DS, 800 mg/160 mg) one tablet twice daily for a planned 10-day course. Terbinafine was also discontinued at this time.

Approximately five days after starting Bactrim, the rash became severely worse. The patient developed intense erythema, tenderness to palpation, severe pruritus, and began to develop clear fluid-filled vesicles and superficial skin peeling. The patient was prescribed oral prednisone 40 mg daily, but the Bactrim was not discontinued at this time.

When symptoms did not improve, he was recommended to go to the hospital. Once at the hospital, oral prednisone and Bactrim were discontinued. 

Key clinical finding: photo-distributed rash

The patient reported that the rash was confined exclusively to sun-exposed areas, including his distal upper extremities, distal lower extremities, and posterior neck. He worked as a commercial driver, spending prolonged hours driving with sun exposure through the driver's side window. His abdomen, chest, back, and genital areas were completely spared.

**Figure 1:**
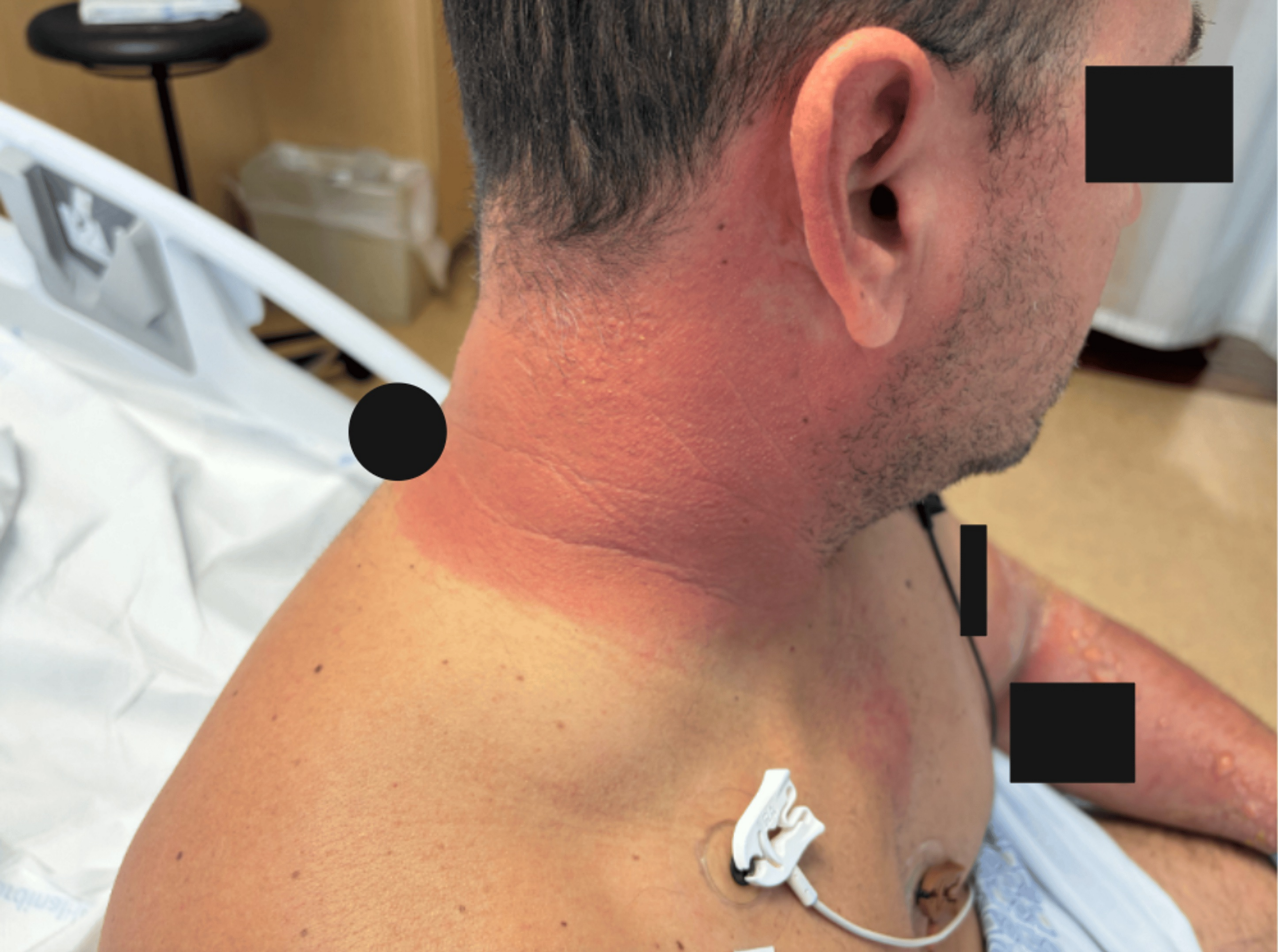
Photo-distributed vesiculobullous rash in the neck and face Right lateral face and neck demonstrating erythema and vesiculation on sun-exposed skin. The eye has been covered to protect patient identity. There is no mucosal involvement. The strict confinement of the rash to sun-exposed areas with sharp demarcation at clothing lines favors phototoxicity over other drug eruptions or infectious processes, which would not show such a precise photo-distributed pattern. Black boxes have been placed over tattoos to protect patient identity. All photographs were obtained with patient consent. The rash was confined to sun-exposed areas (face, neck, distal upper extremities), with complete sparing of covered areas (chest, abdomen, back).

**Figure 2:**
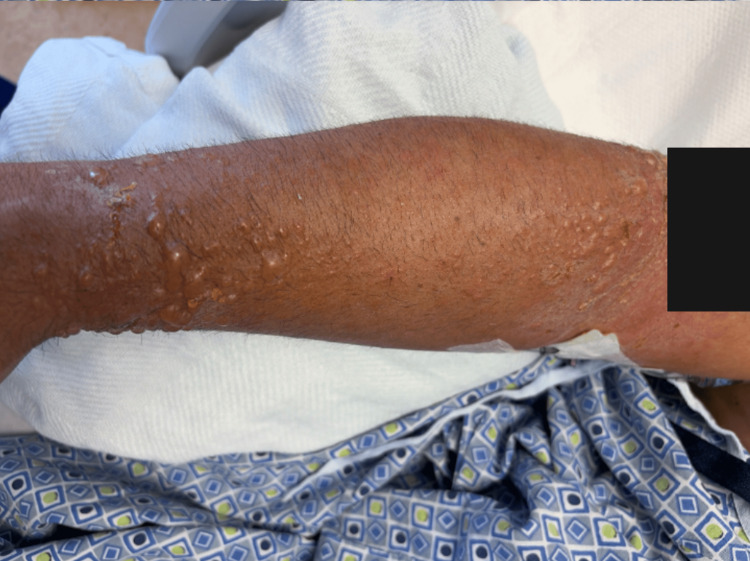
Photo-distributed vesiculobullous rash in right upper extremity. Right upper extremity demonstrating confluent erythema, multiple clear fluid-filled vesicles, and superficial skin peeling. Black boxes have been placed over tattoos to protect patient identity. All photographs were obtained with patient consent. The rash was confined to sun-exposed areas (face, neck, distal upper extremities), with complete sparing of covered areas (chest, abdomen, back).

**Figure 3:**
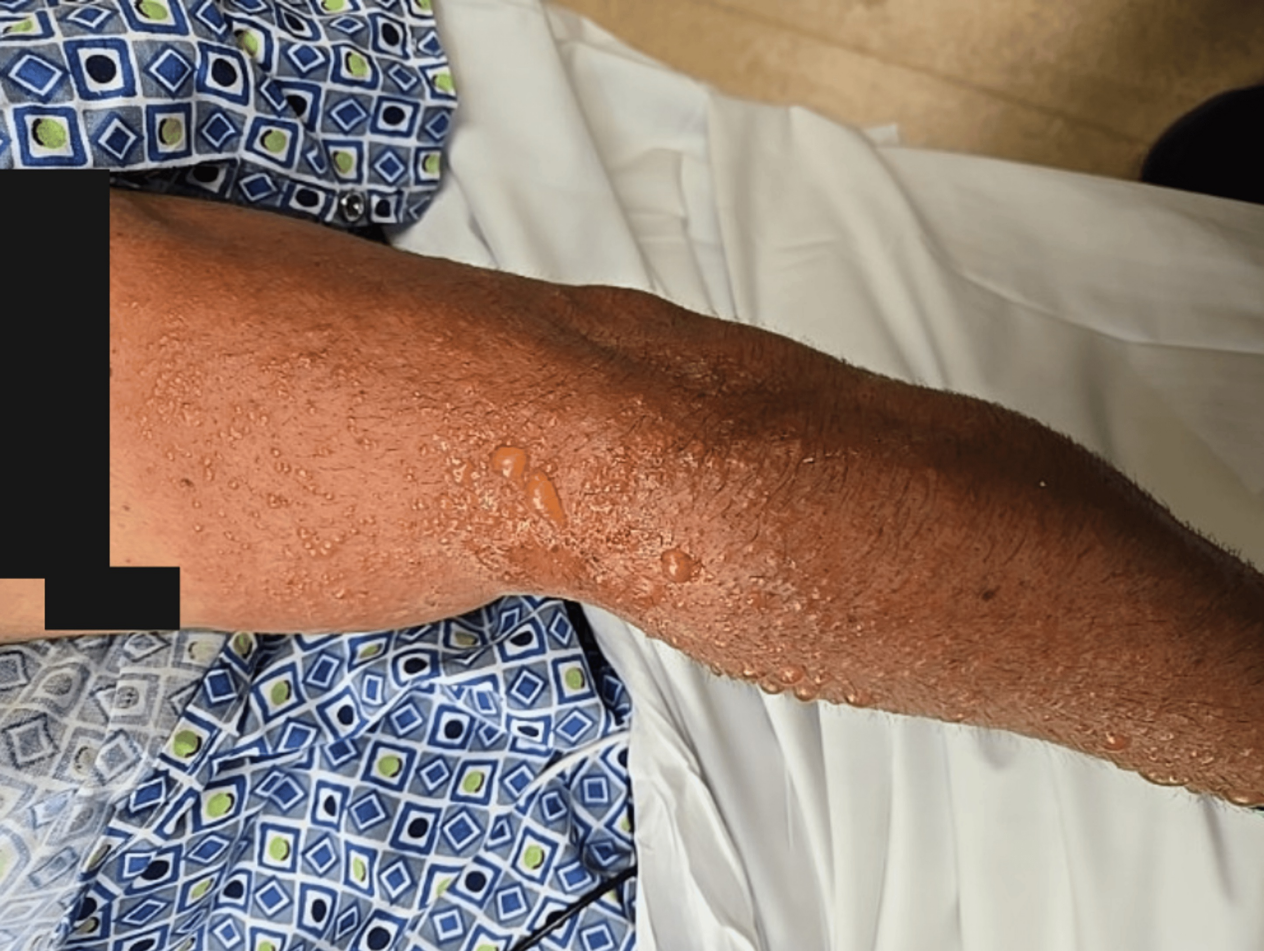
Photo-distributed vesiculobullous rash in left upper extremity. Left upper extremity demonstrating similar findings, confirming bilateral symmetry of the photo-distributed rash. Black boxes have been placed over tattoos to protect patient identity. All photographs were obtained with patient consent. The rash was confined to sun-exposed areas (face, neck, distal upper extremities), with complete sparing of covered areas (chest, abdomen, back).

The photo-distributed rash is shown in Figures 1, 2, and 3.

Hospital admission

The patient presented to the emergency department shortly thereafter with the above symptoms. Upon my evaluation, the patient was afebrile, hemodynamically stable, and without systemic symptoms. There was no mucosal involvement (oral, conjunctival, or genital). Nikolsky's sign was negative. Vital signs were unremarkable.

Physical examination

Dermatological examination revealed a photo-distributed, erythematous, vesiculobullous rash involving the distal arms, distal legs, and posterior neck. The rash was characterized by confluent erythema, multiple clear fluid-filled vesicles and bullae, superficial skin peeling in affected areas, tenderness to palpation, and severe pruritus.

The abdomen, chest, back, and genital areas were completely spared. There was no facial edema, no lymphadenopathy, and no organomegaly.

Diagnostic studies

Initial diagnostic laboratory results are summarized in Table 1. Urinalysis was unremarkable.

**Table 1:** Key laboratory findings on hospital admission The elevated C-reactive protein was nonspecific for infection or inflammation. The normal white blood cell counts and negative blood cultures help argue against cellulitis or systemic infection as the cause of the eruption. SSA - Sjögren's syndrome antigen A; SSB - Sjögren's syndrome antigen B; HIV - human immunodeficiency virus; RPR - rapid plasma reagin; DRESS - drug reaction with eosinophilia and systemic symptoms; ID - infectious disease

Parameter	Patient value	Reference range	Interpretation
Inflammatory markers			
C-reactive protein (CRP)	2.7	0.0 - 0.9 mg/dL	Elevated
Erythrocyte sedimentation rate (ESR)	7	<21 mm/hr	Normal
Autoimmune panel			
Antinuclear antibody (ANA)	Negative	Negative	No autoimmune connective tissue disease
Anti-SSA (Ro) antibody	<0.2	Negative	Negative
Anti-SSB (La) antibody	<0.2	Negative	Negative
Infectious workup			
HIV 1/2 antigen/antibody	Negative	Negative	Negative
RPR (syphilis)	Nonreactive	Nonreactive	Negative
Hepatitis B surface antigen	Negative	Negative	Negative
Hepatitis C antibody	Negative	Negative	Negative
Blood cultures (five days)	No growth	No growth	Negative
Urinalysis	Negative	Negative	No infection
Complete blood count			
White blood cell count	Within normal limits	-	No leukocytosis
Eosinophils	Within normal limits	-	No eosinophilia (DRESS considered unlikely)
Comprehensive metabolic panel			
All parameters	Within normal limits	-	No organ involvement
Infectious disease consult			
ID consultant assessment	Cellulitis and active infection were considered unlikely contributors to the bullous eruption based on clinical presentation	-	Infectious etiology excluded

Timeline summary

The chronological sequence of drug exposures and clinical progression is summarized in Table 2.

**Table 2:** Timeline of Drug Exposure and Clinical Progression This table summarizes the chronological sequence of drug exposures, clinical events, and rash progression from the start of terbinafine therapy (designated as day 0) through hospital admission. Day 0 was defined as the day the patient initiated oral and topical terbinafine. Key observations include the sequential introduction of three photosensitizing medications (terbinafine, ciprofloxacin, and trimethoprim-sulfamethoxazole) and the continued use of Bactrim after prednisone was started on day 18, which explains the lack of initial clinical improvement. Rash resolution occurred only after both medications were discontinued at hospital admission on day 20. PCP - primary care physician; BID - twice daily; DS - double strength

Day	Event	Clinical Status
Day 0	Terbinafine started (topical BID + oral 250 mg daily)	Normal
Day 7	PCP visit – rash worsening; ciprofloxacin started (500 mg BID); terbinafine stopped	Rash present, worsening
Day 13	Ciprofloxacin stopped; Bactrim started (DS BID)	Rash continued
Day 18	Severe worsening; prednisone 40 mg started, Bactrim was not stopped	Vesicles, bullae, skin peeling
Day 20	Hospital admission, oral prednisone and Bactrim were discontinued	Photodistributed rash confirmed

Management and hospital course

All suspected offending medications (terbinafine, ciprofloxacin, trimethoprim-sulfamethoxazole) were discontinued on admission.

The patient was treated with intravenous methylprednisolone 40 mg every eight hours for three days, topical clobetasol ointment applied twice daily to affected areas, diphenhydramine 25 mg intravenously every eight hours as needed for pruritus, and intravenous hydration with normal saline for supportive care.

The patient demonstrated rapid clinical improvement. By hospital day two, erythema had substantially decreased. By hospital day three, erythema had completely resolved, vesicles had significantly decreased, and pain had resolved completely. The patient was clinically ready for discharge on day three.

The patient was discharged home on a prednisone taper (to be completed over approximately two weeks) with instructions for strict sun avoidance and the use of broad-spectrum sunscreen. He was counseled to avoid all photosensitizing medications in the future, particularly terbinafine, fluoroquinolones, and sulfonamides.

An infectious disease consultant evaluated the patient during admission. No infectious etiology was identified. Onychomycosis and cellulitis were considered unlikely based on the clinical presentation, absence of systemic signs of infection, negative laboratory workup (including normal white blood cell count and negative blood cultures), and rapid improvement following discontinuation of antifungal and antibiotic therapy.

A skin biopsy was not performed, as the patient declined due to discomfort, and clinical improvement was rapid. Dermatology consultation was not available at our institution.

## Discussion

This case provides several important learning points regarding the diagnosis and management of severe phototoxic drug eruptions.

Sequential potosensitization is unusual but important to recognize

The most distinctive feature of this case is the sequential exposure to three photosensitizing medications; each associated with progressive clinical worsening. Terbinafine may have initiated the reaction, ciprofloxacin may have exacerbated it, and trimethoprim-sulfamethoxazole may have further worsened it. To our knowledge, sequential photosensitization to three different drug classes in a single patient has rarely been reported.

Sequential photosensitization may occur through cumulative phototoxic injury, where prior exposure to one photosensitizing agent primes the skin for an amplified reaction upon subsequent exposure to another agent. This "priming" effect may be mediated by the accumulation of phototoxic metabolites or by subclinical photodamage that lowers the threshold for reaction to additional photosensitizers.

Terbinafine is a known photosensitizing agent, with case reports describing both phototoxic and photoallergic reactions [10,11]. The onset of rash on day seven of terbinafine therapy in this case is consistent with reported latency periods.

Ciprofloxacin is among the most well-documented photosensitizing fluoroquinolones, with phototoxicity occurring in 1-2% of exposed patients in some series [8,9]. The exacerbation of rash on day six of ciprofloxacin therapy is consistent with this known adverse effect.

Trimethoprim-sulfamethoxazole is also a recognized photosensitizer, though less commonly implicated than fluoroquinolones [1,4]. The severe worsening on day five of Bactrim therapy in this case suggests that the patient's skin may have been primed by prior photosensitizers, resulting in an amplified reaction. Bactrim was continued even after oral prednisone was started on day five, which explains the lack of initial clinical improvement. Rash resolution occurred only after both medications were discontinued at hospital admission.

The diagnostic approach: exclusion of alternatives

Given the broad differential diagnosis for a photo-distributed vesiculobullous rash, we systematically considered alternative conditions. The clinical features that distinguish phototoxic drug eruption from other diagnoses are summarized in Table 3.

**Table 3:** Differential diagnosis of photo-distributed vesiculobullous rash This table summarizes the key clinical features that distinguish phototoxic drug eruption from other conditions that may present with photodistributed or vesiculobullous rashes. The differential diagnosis includes Stevens-Johnson syndrome/toxic epidermal necrolysis (SJS/TEN), DRESS syndrome, systemic and cutaneous lupus erythematosus, drug-induced lupus erythematosus, Sjögren's syndrome, HIV-associated dermatoses, secondary syphilis, viral exanthems, fixed drug eruption, and porphyria cutanea tarda. For each condition, the specific clinical or laboratory feature that makes it less likely in this case is provided. Table 3 is original content created by the authors based on clinical reasoning for this specific patient. It does not reproduce any published material, and no permission is required. Diagnostic certainty is limited by the absence of biopsy and phototesting. DRESS - drug reaction with eosinophilia and systemic symptoms; SSA - Sjögren's syndrome antigen A; SSB - Sjögren's syndrome antigen B; HIV - human immunodeficiency virus; RPR - rapid plasma reagin

Condition	Why less likely
Stevens-Johnson syndrome / toxic epidermal necrolysis	No mucosal involvement, no Nikolsky sign, normal labs, no systemic symptoms, no organ involvement [14]
DRESS syndrome	No eosinophilia, no atypical lymphocytes, normal liver function tests (LFTs), normal creatinine, no fever, no lymphadenopathy [13,15]
Systemic lupus erythematosus / cutaneous lupus	Negative ANA, negative SSA/SSB, no systemic symptoms, no autoantibodies
Drug-induced lupus erythematosus	Negative ANA, no arthralgias, no serositis
Sjögren's syndrome	Negative SSA/SSB, no sicca symptoms (dry eyes, dry mouth)
HIV-associated dermatoses	Negative HIV testing
Secondary syphilis	Negative RPR
Viral exanthem	Negative viral hepatitis panel, negative HIV, no systemic symptoms
Fixed drug eruption	Distribution not typical (would not be limited to sun-exposed areas, would not involve vesicles/bullae)
Porphyria cutanea tarda	No history of liver disease, no family history, no blistering on non-sun-exposed areas, no urine abnormalities
Phototoxic drug eruption	Most likely based on clinical presentation – sun-exposed distribution, vesiculobullous morphology, temporal relationship with three known photosensitizing drugs, rapid improvement after drug withdrawal [1,2,6]

By process of elimination, combined with positive clinical features (photo-distributed rash, vesiculobullous morphology, temporal relationship with known photosensitizing drugs, and rapid improvement following drug withdrawal), the clinical diagnosis of phototoxic drug eruption was established [1,2,6].

The diagnosis of phototoxic drug eruption in this case was made on clinical grounds alone, based on the characteristic photo-distributed rash morphology, temporal relationship with known photosensitizing medications, absence of mucosal involvement, negative Nikolsky sign, and rapid improvement following drug withdrawal and sun avoidance. Histopathological confirmation and phototesting were not available, which limits definitive diagnostic certainty. However, the clinical presentation was highly consistent with phototoxic drug eruption, and alternative diagnoses were systematically excluded.

Role of systemic corticosteroids in severe phototoxicity

The patient received intravenous methylprednisolone followed by an oral prednisone taper, with rapid and complete resolution of symptoms. However, the relative contribution of systemic corticosteroids versus drug discontinuation and sun avoidance cannot be definitively determined. While mild phototoxic reactions can be managed with drug withdrawal, sun avoidance, and supportive care alone, severe vesiculobullous reactions may benefit from systemic corticosteroids to reduce inflammation, prevent secondary infection, and accelerate healing [1,2,8]. The rapid improvement observed in this case likely reflects the combined effect of all three interventions.

The clinical mimics: why recognition matters

Phototoxic drug eruptions can be mistaken for more serious conditions, including SJS/TEN, DRESS syndrome, and cutaneous lupus. This case demonstrates the importance of differentiating these entities because management differs substantially [6,12]. SJS/TEN requires immediate drug withdrawal, intensive care monitoring, and sometimes IVIG or cyclosporine [14]. DRESS syndrome requires prolonged corticosteroid tapers and monitoring for viral reactivation [13,15]. Cutaneous lupus requires immunosuppression and sun protection, but drug withdrawal alone is insufficient. In contrast, phototoxic drug eruption typically resolves with drug withdrawal, sun avoidance, and supportive care, with or without short-course corticosteroids [1,2].

Misdiagnosing phototoxicity as SJS/TEN could lead to unnecessary intensive care admission and interventions. Conversely, misdiagnosing SJS/TEN as phototoxicity could delay life-saving treatment. Therefore, a systematic approach to differential diagnosis is essential.

Limitations

Several limitations warrant acknowledgment. First, a skin biopsy was not performed, as the patient declined due to discomfort, and clinical improvement was rapid. Histopathological confirmation would have strengthened the diagnosis. Second, a dermatology consultation was not available at our institution. Third, phototesting to confirm the diagnosis was not performed. Fourth, the exact duration and intensity of sun exposure could not be quantified. Fifth, the timeline is based on patient recollection, as exact dates were not documented in the electronic medical record. Sixth, no confirmatory testing (KOH preparation, fungal culture, or dermatologic evaluation) was performed prior to the initial diagnosis of ringworm. Despite these limitations, the clinical presentation - including photo-distributed distribution, vesiculobullous morphology, absence of mucosal involvement, negative Nikolsky sign, rapid improvement following drug withdrawal, and systematic exclusion of infectious, autoimmune, and other drug-induced conditions - is most consistent with phototoxic drug eruption [6,12].

Clinical relevance

For clinicians, this case emphasizes several practical takeaways. First, maintain a high index of suspicion for phototoxic drug eruptions in patients presenting with photo-distributed rashes, particularly those with occupational sun exposure [1,2]. Second, recognize that multiple photosensitizing medications can have additive or synergistic effects, as sequential prescribing may worsen a pre-existing reaction [4,6]. Third, terbinafine, fluoroquinolones, and sulfonamides are all potential photosensitizers [1,4,8-11], so a thorough medication history should include recent antifungal and antibiotic exposures. Fourth, systematic exclusion of alternative diagnoses such as infection, autoimmunity, SJS/TEN, and DRESS is essential when confirmatory testing is not available [6,12]. Fifth, severe phototoxic reactions may benefit from short-course systemic corticosteroids in addition to drug withdrawal and sun protection [1,2,8]. The rapid improvement observed in this case likely reflects the combined effect of drug discontinuation, sun avoidance, and corticosteroids.

## Conclusions

This case of severe phototoxic vesiculobullous eruption following sequential exposure to terbinafine, ciprofloxacin, and trimethoprim-sulfamethoxazole illustrates the importance of recognizing phototoxic drug eruptions in patients presenting with photo-distributed rashes, particularly when multiple photosensitizing medications have been prescribed sequentially. The systematic exclusion of infectious, autoimmune, and other drug-induced conditions is essential for accurate diagnosis when biopsy and specialty consultation are not available. Clinicians should maintain awareness that terbinafine, fluoroquinolones, and sulfonamides are all potential photosensitizers, and sequential exposure may produce a severe, amplified reaction. Prompt recognition, immediate withdrawal of offending agents, sun avoidance, and appropriate use of systemic corticosteroids can lead to rapid and complete recovery. For patients with chronic occupational sun exposure, such as commercial drivers, future treatment decisions involving potentially photosensitizing medications should be approached cautiously, with consideration of safer alternatives when available and strict photoprotection measures (including broad-spectrum sunscreen and protective clothing) when photosensitizers cannot be avoided.
